# Comparison of bariatric surgery and community weight management for idiopathic intracranial hypertension in a multicenter retrospective cohort study

**DOI:** 10.1038/s41598-025-97081-5

**Published:** 2025-04-22

**Authors:** Rasha Tarek Mirdad, Mahmoud M. Morsy, Ahmed Y. Azzam, Alsaleem Mohammed Abadi, Abdullah A. Dalboh, Nasser A. Alsabaani, Waleed A. Aldhabaan, Moutasem S. Aboonq, Muhammed Amir Essibayi, Mohamed D. Morsy, David J. Altschul

**Affiliations:** 1 Department of Surgery, College of Medicine, King Khalid University, Abha, Saudi Arabia; 2 October 6 University Hospital, October 6 University, Giza, Egypt; 3 Montefiore-Einstein Cerebrovascular Research Lab, Albert Einstein College of Medicine, Bronx, NY USA; 4 Family and Community Medicine Department, College of Medicine, King Khalid University, Abha, Saudi Arabia; 5 Surgery Department, Faculty of Medicine, King Khalid University, Abha, Saudi Arabia; 6 Department of Ophthalmology, College of Medicine, King Khalid University, Abha, Saudi Arabia; 7 Department of Clinical Physiology, College of Medicine, Taibah University, Al-Madinah Al-Munawwarah, Saudi Arabia; 8 Department of Neurological Surgery, Montefiore Medical Center, Albert Einstein College of Medicine, Bronx, NY USA; 9 Department of Clinical Physiology, College of Medicine, King Khalid University, Abha, Saudi Arabia

**Keywords:** Idiopathic intracranial hypertension, Pseudotumor cerebri, Weight loss, Bariatric surgery, Obesity, Neuroscience, Medical research, Neurology

## Abstract

Idiopathic Intracranial Hypertension (IIH) is a neurological disorder characterized by elevated intracranial pressure without definitive etiology, primarily affecting young, obese women. This study aimed to compare the efficacy of bariatric surgery versus conventional community weight management in treating IIH. We conducted a retrospective cohort study in IIH patients undergoing bariatric procedures versus conventional weight loss interventions. Propensity score matching was employed to balance study groups. Outcomes were assessed at 3, 6, 12, and 24 months, including papilledema, headache, visual symptoms, and therapeutic interventions. Bariatric surgery demonstrated superior outcomes compared to community weight management. Papilledema incidence was consistently lower in the bariatric group (RR = 0.591 at 24 months, *p* = 0.0001). Headache prevalence and visual symptoms were also reduced in the surgical group. Acetazolamide dose was lower in bariatric patients, starting at 12 and 24 months. Subgroup analysis of different bariatric procedures showed comparable efficacy. Body mass index reduction was significantly greater in the bariatric group throughout the follow-up period. This study provides evidence supporting the efficacy of bariatric surgery in managing IIH, with superior outcomes across multiple parameters compared to conventional weight management. The sustained improvements in papilledema, headache, and visual symptoms, coupled with for the reduction in pharmacological intervention dose, suggest that bariatric surgery may offer a more definitive solution for IIH patients with concurrent obesity. Further research is needed to develop evidence-based guidelines for patient selection and optimize post-operative care protocols.

## Introduction

Idiopathic Intracranial Hypertension (IIH) is a neurological disorder characterized by elevated intracranial pressure (ICP) in the absence of identifiable etiology^1^. This condition predominantly affects young, obese women of childbearing age, with an estimated incidence of 0.9 per 100,000 in the general population, rising to 19 per 100,000 in women with a body mass index (BMI) exceeding 30 kg/m^2^^2^.The pathophysiology of IIH remains enigmatic, with proposed mechanisms including abnormalities in cerebrospinal fluid (CSF) dynamics, cerebral venous sinus stenosis, and adipose tissue dysfunction^3^. The clinical presentation typically includes headaches, papilledema, and visual disturbances, with the potential for permanent vision loss if left untreated^4^.

The current standard of care for IIH encompasses a multifaceted approach, with weight loss serving as the cornerstone of management. Lifestyle modifications aimed at reducing body weight by 5–10% have demonstrated efficacy in alleviating symptoms and improving visual outcomes^5^.Pharmacological interventions, primarily acetazolamide, a carbonic anhydrase inhibitor, have shown promise in reducing ICP and improving visual field function^6^. The Idiopathic Intracranial Hypertension Treatment Trial (IIHTT) provided robust evidence supporting acetazolamide’s efficacy, demonstrating significant improvements in visual field function and quality of life measures compared to placebo^7^. Additional therapeutic modalities include topiramate, which offers the dual benefit of ICP reduction and weight loss, and CSF diversion procedures such as ventriculoperitoneal or lumboperitoneal shunting for refractory cases^8^.

Despite these established interventions, the management of IIH remains challenging, with significant limitations in current approaches. Long-term adherence to lifestyle modifications and pharmacotherapy is often suboptimal, with weight recidivism being a common issue^9^. Moreover, the side effect profile of acetazolamide, including paresthesias, dysgeusia, and metabolic acidosis, can lead to treatment discontinuation in a subset of patients^10^. The efficacy of CSF diversion procedures is hampered by high failure and revision rates, necessitating repeated surgical interventions^11^. These limitations underscore the need for more definitive and sustainable treatment options, particularly for patients with severe, refractory IIH.

In this context, bariatric surgery has emerged as a promising therapeutic avenue for IIH management, especially in patients with concurrent morbid obesity. The dramatic and sustained weight loss achieved through bariatric procedures offers a potential mechanism for long-term ICP reduction and symptom resolution^12^. Recent studies have reported encouraging outcomes, with significant improvements in papilledema, headache severity, and overall quality of life following bariatric interventions^13^. Ottridge et al. demonstrated resolution or improvement of papilledema in 90% of IIH patients undergoing bariatric surgery, with concomitant reductions in intracranial pressure and headache severity^14^. These findings suggest that bariatric surgery may offer a more definitive solution for carefully selected IIH patients, addressing both the metabolic dysfunction associated with obesity and the elevated ICP characteristic of the condition.

Our study aims to further elucidate the comparative efficacy of bariatric surgery versus conventional community weight management approaches in the treatment of IIH. Utilizing the TriNetX database, a global federated network of electronic health records^15^, we conducted a retrospective cohort study to analyze outcomes in IIH patients undergoing bariatric procedures compared to those managed with traditional weight loss interventions. We aim to provide real-world evidence to inform clinical decision-making and potentially reshape treatment paradigms for IIH. By leveraging a large-scale, diverse patient population, we aim to offer insights into the long-term efficacy, safety, and potential cost-effectiveness of bariatric surgery as a management strategy for IIH, particularly in cases refractory to conventional therapies.

## Methods

We leveraged data from the comprehensive TriNetX Research Network, encompassing over 200 million electronic health records from more than 75 healthcare organizations, predominantly in the United States^15^. The dataset provides rich patient-level information, including demographics, diagnoses, treatments, procedures, and outcomes, coded using standard medical classification systems such as ICD-10 and CPT. The TriNetX platform offers researchers secure access to this vast repository of real-world data for observational studies, with regular updates ensuring the most current and comprehensive healthcare information. We conducted a retrospective analysis of TriNetX data from 2009 to September 2024 (the available data timeframe on the dataset), focusing on patients diagnosed with IIH. Exclusion criteria encompassed individuals with other known causes of elevated intracranial pressure, including primary brain tumors, secondary brain metastases, cerebral arteriovenous malformations, malignant hypertension (primary and secondary), meningitis, traumatic elevated intracranial pressure, and venous sinus thrombosis.

To ensure well-balanced study groups, we employed propensity score matching based on age, sex, race, ethnicity, and baseline body mass index (BMI) at the time of weight loss management initiation, either through bariatric surgery or conventional weight management approaches. Our analysis examined outcomes at various follow-up intervals (3, 6, 12, and 24 months), assessing key indicators such as papilledema, increased intracranial pressure (ICP), headache, optic atrophy, blindness, pulsatile tinnitus, abducent nerve palsy, diplopia, refractory IIH status, visual discomfort, and visual field defects. Additionally, we evaluated therapeutic intervention rates, including spinal punctures, CSF shunting, and optic nerve sheath fenestration (ONSF), as well as the continued use of acetazolamide as the primary treatment.

All of the used methods in this retrospective cohort study were carried out in accordance with relevant guidelines and regulations, including the Declaration of Helsinki and Good Clinical Practice guidelines for retrospective database research. Our study was determined to be exempt from review by the Albert Einstein College of Medicine Institutional Review Board as it does not constitute human subjects research according to 45 CFR 46.104(d)(4); we also confirm that the study protocol was approved by the Albert Einstein College of Medicine Institutional Review Board with status of waiver as the same condition for both the protocol and study implementation under 45 CFR 46.104(d)(4). Also, the requirement for individual informed consent was waived as this study analyzed only de-identified data from the TriNetX Research Network, where all participating healthcare organizations have established data use agreements and appropriate patient privacy protocols in accordance with the Health Insurance Portability and Accountability Act (HIPAA) regulations. The utilized data was obtained from TriNetX platform without direct access to individual data level, the accessibility to data analysis shall be obtained from the platform directly.

IIH diagnosis was established based on ICD-10 coding (G93.2) in the TriNetX database, which follows the modified Dandy criteria including: (1) signs and symptoms of increased intracranial pressure; (2) no localizing neurological findings except abducens nerve palsy; (3) CSF opening pressure ≥ 25 cmH2O with normal CSF composition; (4) normal neuroimaging (MRI/MRV) except for empty sella turcica, optic nerve sheath distention, and smooth-walled venous sinus stenosis; and (5) no other identified cause of intracranial hypertension. The assessment of papilledema was based on documented ophthalmological examinations in the electronic health records, including fundoscopic examination findings and, where available, optical coherence tomography (OCT) measurements. Visual field defects were evaluated through documented automated perimetry results, though the specific testing protocols varied across participating institutions due to the retrospective nature of the study.

### Statistical analysis

The TriNetX platform is equipped with a suite of powerful analytical tools, leveraging programming languages such as Java, R, and Python, which enabled the researchers to efficiently query and analyze the comprehensive dataset to extract meaningful insights^15^. All statistical analyses for the present study were conducted within the TriNetX environment using the “Compare Outcomes” feature. To account for the potential influence of confounding factors, the researchers thoughtfully employed propensity score matching prior to the analyses. This involved a 1:1 matching approach, utilizing nearest neighbor matching without replacement and a caliper set at 0.1 times the standard deviation. TriNetX’s proprietary algorithms derive propensity scores through logistic regression, drawing upon matrices of covariates with randomized row order to enhance the robustness of the matching process. The criterion for statistical significance was set at a p-value less than 0.05. This threshold was chosen to balance the need for robust evidence while allowing for the detection of potentially meaningful effects, acknowledging the inherent complexities and nuances present in real-world data^15^.

### Baseline demographics

Our cohort initially comprised 1,345 bariatric surgery patients and 1,705 community weight management patients, as listed in Table 1. Prior to propensity score matching, significant differences were observed between the groups across multiple variables (*p* < 0.00001). The bariatric surgery group was older (mean age 37.5 vs. 32.3 years) and had a higher proportion of females (90.18% vs. 83.75%). After propensity score matching, 993 patients were included in each group, resulting in well-balanced cohorts with no statistically significant differences in baseline characteristics. In the matched cohorts, mean age was comparable (35.4 vs. 35.2 years, *p* = 0.7374), as was the gender distribution (88.62% vs. 89.124% female, *p* = 0.7213). Racial and ethnic composition was similar between groups, with non-Hispanic or Latino individuals comprising the majority (73.82% vs. 74.8%, *p* = 0.6075). The prevalence of associated systemic diseases was also balanced post-matching, with endocrine, nutritional, and metabolic diseases being the most common in both groups (74.01% vs. 75.529%, *p* = 0.4383), followed by musculoskeletal (58% vs. 59.41%, *p* = 0.5234) and gastrointestinal disorders (54.783% vs. 55.891%, *p* = 0.6195).

**Table 1:** Baseline demographics of the included cohort.

Variable	Before propensity score matching		*P*-value	After propensity score matching		*P*-value
	Bariatric surgery	Community weight management		Bariatric surgery	Community weight management	
Total Patients, n	1345	1705	< 0.00001	993	993	0.7374
Mean Age (years)	37.5	32.3	< 0.00001	35.4	35.2	0.7374
Standard Deviation	12	12.4	< 0.00001	10.9	13.1	0.7374
Gender						
Female, n (%)	1213 (90.18%)	1428 (83.75%)	< 0.00001	880 (88.62%)	885 (89.124%)	0.7213
Male, n (%)	91 (6.7%)	188(11%)	< 0.00001	75 (7.5%)	70 (7.04%)	0.6663
Unknown, n (%)	41 (3.04%)	89 (5.22%)	0.0032	38 (3.827%)	38 (3.827%)	0.999
Race						
Non-Hispanic or Latino, n (%)	977 (72.6%)	1243 (72.9%)	0.8709	733	743 (74.8%)	0.6075
Unknown, n (%)	248 (18.4%)	234 (13.7%)	0.0004	142 (14.3.%)	132 (13.293)	0.5153
Hispanic or Latino, n (%)	120 (8.9%)	228 (13.3%)	0.0001	105 (10.57%)	105 (10.57%)	0.999
White, n (%)	819 (60.8%)	1040 (60.9%)	0.953	605 (60.9%)	613 (61.732)	0.7124
Black or African American, n (%)	234 (17.3%)	315 (18.47%)	0.44	186 (18.7%)	190 (19.134%)	0.8188
Other Race, n (%)	36 (2.6%)	69 (4%)	0.03	32 (3.2%)	35 (3.5%)	0.7093
Asian, n (%)	36 (2.6%)	41 (2.4%)	0.63	24 (2.4%)	19 (1.9%)	0.4408
American Indian, n (%)	10 (0.7%)	11 (0.6%)	0.7444	10 (1%)	10 (1%)	0.999
Native Hawaiian or Other Pacific, n (%)	10 (0.7%)	11 (0.6%)	0.7444	10 (1%)	10 (1%)	0.999
Associated systemic diseases						
Endocrine, nutritional and metabolic diseases, n (%)	928 (68.9%)	1278 (74.956%)	0.0003	735 (74.01%)	750 (75.529%)	0.4383
Diseases of musculoskeletal system, n (%)	695 (51.673%)	1077 (63.167%)	< 0.0001	576 (58%)	590 (59.41%)	0.5234
Diseases of gastrointestinal and digestive system, n (%)	711 (52.86%)	908 (53.25)	0.8292	544 (54.783%)	555 (55.891%)	0.6195
Diseases of eye and adnexa, n (%)	594 (44.16%)	1083(63.51%)	< 0.0001	525 (52.87%)	522 (52.568%)	0.8927
Diseases of respiratory system, n (%)	605(44.98%)	1065(62.463%)	< 0.0001	523 (52.669%)	536 (53.97%)	0.5587
Diseases of circulatory system, n (%)	329 (24.46%)	508(29.795%)	0.001	277 (27.89%)	279 (28.097%)	0.9204

## Results

### Outcomes analysis

Our results implicated significant differences in outcomes between bariatric surgery and community weight management groups across various follow-up periods as demonstrated in Table 2. Notably, papilledema incidence was consistently lower in the bariatric surgery group, with risk ratios ranging from 0.5 (95% CI: 0.328–0.763, *p* = 0.001) at three months to 0.591 (95% CI: 0.45–0.775, *p* = 0.0001) at 24 months. Headache prevalence also showed a generally favorable trend for the bariatric surgery group, with statistically significant reductions at 3, 12, and 24 months (RR: 0.871, 0.885, and 0.9, respectively; all *p* < 0.05). Interestingly, blindness rates were initially similar between groups but diverged at 12 months, with the bariatric surgery group showing a higher incidence (3.11% vs. 1.60%, RR: 1.882, 95% CI: 1.052–3.368, *p* = 0.0301). Visual discomfort and visual field defects were less prevalent in the bariatric surgery group, reaching statistical significance at 12 and 24 months (RR: 0.565 and 0.574, respectively; both *p* < 0.05). Acetazolamide use was lower in the bariatric surgery group throughout the follow-up period, with significant differences emerging at 12 and 24 months (RR: 0.815 and 0.801, respectively; both *p* < 0.05). Notably, no ONSF procedures were performed in the bariatric surgery group, compared to a consistent 0.97–1.01% rate in the community weight management group (*p* = 0.0015 at all-time points).

**Table 2:** Results of the outcomes over longitudinal follow-up time points.

Outcome	Follow-up duration	Bariatric surgery	Community weight management	Risk difference	Risk ratio	95% confidence interval	*P*-value
Papilledema	3 months	3.12%	6.24%	− 3.12%	0.5	0.328–0.763	0.001
	6 months	5.04%	9.32%	− 4.29%	0.54	0.392–0.744	0.0001
	12 months	6.42%	11.19%	− 4.77%	0.574	0.429–0.767	0.0001
	24 months	7.29%	12.35%	− 5.06%	0.591	0.45–0.775	0.0001
Increased ICP	3 months	1.01%	1.01%	0	1	0.418–2.392	0.999
	6 months	0.93%	0.93%	0	1	0.418–2.393	0.999
	12 months	1.36%	0.97%	0.39%	1.4	0.625–3.137	0.4115
	24 months	1.75%	1.27%	0.49%	1.385	0.682–2.811	0.3655
Headache	3 months	30.60%	35.14%	− 4.35%	0.871	0.768–0.988	0.03
	6 months	38.15%	42.07%	− 3.92%	0.9	0.818–1.006	0.0642
	12 months	44.16%	49.90%	− 5.74%	0.885	0.807–0.97	0.0091
	24 months	49.03%	54.48%	− 5.45%	0.9	0.828–0.979	0.0135
Optic atrophy	3 months	1.01%	1.01%	0	1	0.418–2.392	0.999
	6 months	0.93%	0.93%	0	1	0.418–2.393	0.999
	12 months	0.97%	1.46%	− 0.49%	0.667	0.301–1.477	0.3144
	24 months	0.97%	1.95%	− 0.97%	0.5	0.235–1.063	0.0659
Blindness	3 months	1.31%	1.11%	0.00201	1.18	0.532–2.62	0.68
	6 months	1.86%	1.31%	0.56%	1.429	0.725–2.813	0.299
	12 months	3.11%	1.60%	1.46%	1.882	1.052–3.368	0.0301
	24 months	3.79%	2.43%	1.36%	1.56	0.951–2.558	0.0754
Pulsatile tinnitus	3 months	1.01%	1.01%	0	1	0.418–2.392	0.999
	6 months	0.93%	0.93%	0	1	0.418–2.393	0.999
	12 months	0.97%	1.46%	− 0.49%	0.667	0.301–1.477	0.3144
	24 months	0.97%	1.84%	− 0.88%	0.52	0.246–1.126	0.0923
Abducent nerve palsy	3 months	1.01%	1.01%	0	1	0.418–2.392	0.999
	6 months	0.93%	0.93%	0	1	0.418–2.393	0.999
	12 months	0.97%	0.97%	0	1	0.418–2.392	0.999
	24 months	0.97%	0.97%	0	1	0.418–2.392	0.999
Diplopia	3 months	1.01%	1.01%	0	1	0.418–2.392	0.999
	6 months	1.11%	0.93%	0.19%	1.2	0.521–2.766	0.6682
	12 months	1.27%	1.27%	0	1	0.466–2.147	0.999
	24 months	1.36%	1.75%	− 0.39%	0.778	0.389–1.555	0.476
Refractory IIH	3 months	29.4	32.22	− 2.82%	0.913	0.8–1.04	0.1736
	6 months	36.85%	38.81%	− 1.96%	0.95	0.852–1.058	0.3497
	12 months	42.89%	46.40%	− 3.50%	0.925	0.84–1.018	0.1103
	24 months	47.37%	50.68%	− 3.31%	0.935	0.856–1.021	0.1336
Visual discomfort and visual field defects	3 months	1.41%	2.11%	− 0.71%	0.66	0.341–1.304	0.2326
	6 months	1.68%	2.89%	− 1.21%	0.581	0.327–1.031	0.0603
	12 months	2.53%	4.48%	− 1.95%	0.565	0.352–0.907	0.0164
	24 months	3.02%	5.25%	− 2.24%	0.574	0.372–0.885	0.0108
CSF leak	3 months	1.01%	1.01%	0	1	0.418–2.392	0.999
	6 months	0.93%	0.93%	0	1	0.418–2.393	0.999
	12 months	0.97%	0.97%	0	1	0.418–2.392	0.999
	24 months	0.97%	0.97%	0	1	0.418–2.392	0.999
Therapeutic spinal puncture rate	3 months	1.01%	1.01%	0	1	0.418–2.392	0.999
	6 months	0.93%	0.93%	0	1	0.418–2.393	0.999
	12 months	1.27%	0.97%	0.29%	1.3	0.573–2.951	0.5293
	24 months	1.56%	1.17%	0.39%	1.33	0.634–2.804	0.4466
CSF shunting rate	3 months	1.01%	1.01%	0	1	0.418–2.392	0.999
	6 months	0.93%	0.93%	0	1	0.418–2.393	0.999
	12 months	1.16%	0.97%	0.20%	1.2	0.521–2.765	0.6681
	24 months	1.36%	0.97%	0.39%	1.4	0.625–3.137	0.4115
ONSF rate	3 months	0%	1.01%	− 1.01%	N/A	N/A	0.0015
	6 months	0%	0.93%	− 0.93%	N/A	N/A	0.0015
	12 months	0%	0.97%	− 0.97%	N/A	N/A	0.0015
	24 months	0%	0.97%	− 0.97%	N/A	N/A	0.0015
Acetazolamide use	3 months	9.87%	11.07%	− 1.20%	0.891	0.689–1.153	0.37
	6 months	11.57%	14.27%	− 2.52%	0.824	0.661–1.026	0.0831
	12 months	14.59%	17.90%	− 3.31%	0.815	0.669–0.993	0.0421
	24 months	16.05%	20.04%	− 3.99%	0.801	0.665–0.964	0.0187

### Subgroup comparison according to each bariatric procedure

#### Sleeve gastrectomy versus adjustable gastric banding

In comparing sleeve gastrectomy to adjustable gastric banding, our results revealed no statistically significant differences in most outcomes across the follow-up periods (Table 3). Papilledema rates were similar between the two procedures, with risk ratios ranging from 0.813 (95% CI: 0.396–1.669, *p* = 0.5711) at three months to 1.033 (95% CI: 0.637–1.677, *p* = 0.8944) at 24 months. Headache prevalence showed a slight, non-significant trend favoring adjustable gastric banding, with risk ratios consistently close to 1.0 (range: 1.014–1.096, all *p* > 0.05). Refractory IIH rates were comparable between groups throughout the follow-up period, with the largest difference observed at three months (RR: 1.092, 95% CI: 0.885–1.346, *p* = 0.41). Notably, no cases of increased intracranial pressure were reported in either group at three months, and no ONSF procedures were performed in either group throughout the study period. Acetazolamide use was initially identical between groups (9.03% at three months) but showed a non-significant trend towards lower usage in the sleeve gastrectomy group at 24 months (13.77% vs. 17.16%, RR: 0.803, 95% CI: 0.589–1.094, *p* = 0.1634). Interestingly, pulsatile tinnitus and abducent nerve palsy were observed exclusively in the adjustable gastric banding group at certain time points, though the overall incidence remained low (2.26–2.32%).

**Table 3:** Sleeve gastrectomy versus adjustable gastric banding outcomes comparison.

Outcome	Follow-up duration	Sleeve gastrectomy	Adjustable gastric banding	Risk difference	Risk ratio	95% confidence interval	*P*-value
Papilledema	3 months	2.93%	3.61%	− 0.67%	0.813	0.396–1.669	0.5711
	6 months	4.17%	3.47%	0.69%	1.2	0.613–2.35	0.5944
	12 months	5.42%	5.87%	− 0.451	0.923	0.538–1.582	0.7709
	24 months	7.00%	6.77%	0.23%	1.033	0.637–1.677	0.8944
Increased ICP	3 months	0	0	N/A	N/A	N/A	N/A
	6 months	2.32%	2.32%	0	1	0.42–2.378	0.999
	12 months	2.26%	2.26%	0	1	0.42–2.379	0.999
	24 months	2.26%	2.26%	0	1	0.42–2.379	0.999
Headache	3 months	30.93%	28.22%	2.71%	1.096	0.894–1.343	0.377
	6 months	37.04%	35.19%	1.85%	1.053	0.881–1.257	0.571
	12 months	42.89%	41.76%	1.13%	1.027	0.881–1.198	0.7339
	24 months	47.40%	46.73%	0.68%	1.014	0.882–1.167	0.84
Optic Atrophy	3 months	2.26%	2.26%	0	1	0.42–2.379	0.999
	6 months	2.32%	2.32%	0	1	0.42–2.378	0.999
	12 months	2.26%	2.26%	0	1	0.42–2.379	0.999
	24 months	2.26%	2.26%	0	1	0.42–2.379	0.999
Blindness	3 months	2.26%	2.26%	0	1	0.42–2.379	0.999
	6 months	2.32%	2.32%	0	1	0.42–2.378	0.999
	12 months	2.26%	3.39%	− 1.13%	0.667	0.303–1.468	0.3104
	24 months	2.71%	4.29%	− 1.58%	0.632	0.31–1.285	0.2006
Pulsatile tinnitus	3 months	0	0	N/A	N/A	N/A	N/A
	6 months	0	2.32%	− 2.32%	N/A	N/A	0.0015
	12 months	2.26%	2.26%	0	1	0.42–2.379	0.999
	24 months	2.26%	2.26%	0	1	0.42–2.379	0.999
Abducent nerve palsy	3 months	0	2.26%	− 2.26%	N/A	N/A	0.0015
	6 months	0	2.32%	− 2.32%	N/A	N/A	0.0015
	12 months	0	2.26%	− 2.26%	N/A	N/A	0.0015
	24 months	2.26%	2.26%	0	1	0.42–2.379	0.999
Diplopia	3 months	2.26%	2.26%	0	1	0.42–2.379	0.999
	6 months	2.32%	2.32%	0	1	0.42–2.378	0.999
	12 months	2.26%	2.26%	0	1	0.42–2.379	0.999
	24 months	2.26%	2.26%	0	1	0.42–2.379	0.999
Refractory IIH	3 months	29.57%	27.09%	2.48%	1.092	0.885–1.346	0.41
	6 months	35.88%	33.57%	2.32%	1.069	0.89–1.284	0.4749
	12 months	41.54%	40.63%	0.90%	1.022	0.873–1.197	0.7847
	24 months	46.05%	45.15%	0.90%	1.02	0.883–1.178	0.7873
Visual discomfort and visual field defects	3 months	2.26%	2.26%	0	1	0.42–2.379	0.999
	6 months	2.32%	2.32%	0	1	0.42–2.378	0.999
	12 months	2.26%	2.94%	− 0.68%	0.769	0.341–1.736	0.5262
	24 months	3.16%	3.61%	− 0.45%	0.875	0.432–1.771	0.7103
CSF leak	3 months	2.26%	2.26%	0	1	0.42–2.379	0.999
	6 months	0.00%	2.32%	− 2.32%	N/A	N/A	0.0015
	12 months	2.26%	2.26%	0%	1	0.42–2.379	0.999
	24 months	2.26%	2.26%	0%	1	0.42–2.379	0.999
Therapeutic spinal puncture rate	3 months	2.26%	2.26%	0	1	0.42–2.379	0.999
	6 months	2.32%	2.32%	0	1	0.42–2.378	0.999
	12 months	2.26%	2.26%	0%	1	0.42–2.379	0.999
	24 months	2.26%	2.26%	0%	1	0.42–2.379	0.999
CSF shunting rate	3 months	0	2.26%	− 2.26%	N/A	N/A	0.0015
	6 months	2.32%	2.32%	0	1	0.42–2.378	0.999
	12 months	2.26%	2.26%	0%	1	0.42–2.379	0.999
	24 months	2.26%	2.26%	0%	1	0.42–2.379	0.999
ONSF rate	3 months	0	0	N/A	N/A	N/A	N/A
	6 months	0	0	N/A	N/A	N/A	N/A
	12 months	0	0	N/A	N/A	N/A	N/A
	24 months	0	0	N/A	N/A	N/A	N/A
Acetazolamide use	3 months	9.029	9.03%	0%	1	0.658–1.519	0.999
	6 months	9.03%	8.80%	0.23%	1.026	0.67–1.572	0.905
	12 months	12.42%	14.45%	− 2.03%	0.859	0.614–1.202	0.3752
	24 months	13.77%	17.16%	− 3.39%	0.803	0.589–1.094	0.1634

#### Roux-en-Y gastric bypass (RYGB) versus sleeve gastrectomy

Comparison of RYGB and sleeve gastrectomy revealed comparable outcomes for most measures throughout the follow-up period (Table 4). Papilledema rates were identical between groups until 24 months, where a non-significant difference emerged (5.03% vs. 5.53%, RR: 0.909, 95% CI: 0.395–2.092, *p* = 0.8226). Headache prevalence showed minor fluctuations, with RYGB demonstrating a non-significant trend towards higher rates at later time points (24 months: 50.25% vs. 45.73%, RR: 1.099, 95% CI: 0.895–1.349, *p* = 0.3665). Refractory IIH rates were similar, with the largest non-significant difference observed at six months (40.20% vs. 34.17%, RR: 1.176, 95% CI: 0.91–1.521, *p* = 0.2133). Notably, increased intracranial pressure was exclusively observed in the RYGB group at three months (5.03%, *p* = 0.0014), while CSF leak was only reported in the sleeve gastrectomy group across all time points (5.03%, *p* = 0.0014 for each interval). Pulsatile tinnitus occurred solely in the RYGB group at 12 and 24 months (5.03%, *p* = 0.0014 for both). Acetazolamide use was consistently higher in the RYGB group, reaching statistical significance at six months (17.09% vs. 10.05%, RR: 1.7, 95% CI: 1.015–2.849, *p* = 0.0404) and 24 months (23.12% vs. 14.07%, RR: 1.643, 95% CI: 1.072–2.517, *p* = 0.0204). Neither group went for CSF shunting or ONSF procedures throughout the cohort’s timeline.

**Table 4:** RYGB versus sleeve gastrectomy outcomes comparison.

Outcome	Follow-up duration	RYGB	Sleeve gastrectomy	Risk difference	Risk ratio	95% confidence interval	*P*-value
Papilledema	3 months	5.03%	5.03%	0	1	0.426–2.35	0.999
	6 months	5.03%	5.03%	0	1	0.426–2.35	0.999
	12 months	5.03%	5.03%	0	1	0.426–2.35	0.999
	24 months	5.03%	5.53%	− 0.50%	0.909	0.395–2.092	0.8226
Increased ICP	3 months	5.03%	0	5.03%	N/A	N/A	0.0014
	6 months	5.03%	5.03%	0	1	0.426–2.35	0.999
	12 months	5.03%	5.03%	0	1	0.426–2.35	0.999
	24 months	5.03%	5.03%	0	1	0.426–2.35	0.999
Headache	3 months	31.16%	33.67%	− 2.51%	0.925	0.697–1.229	0.5923
	6 months	41.21%	34.67%	6.53%	1.188	0.923–1.53	0.1793
	12 months	44.22%	41.21%	3.02%	1.073	0.855–1.348	0.5432
	24 months	50.25%	45.73%	4.52%	1.099	0.895–1.349	0.3665
Optic atrophy	3 months	5.03%	0	5.03%	N/A	N/A	0.0014
	6 months	5.03%	5.03%	0	1	0.426–2.35	0.999
	12 months	5.03%	5.03%	0	1	0.426–2.35	0.999
	24 months	5.03%	5.03%	0	1	0.426–2.35	0.999
Blindness	3 months	5.03%	5.03%	0	1	0.426–2.35	0.999
	6 months	5.03%	5.03%	0	1	0.426–2.35	0.999
	12 months	5.03%	5.03%	0	1	0.426–2.35	0.999
	24 months	5.03%	5.03%	0	1	0.426–2.35	0.999
Pulsatile tinnitus	3 months	0	0	N/A	N/A	N/A	N/A
	6 months	0	0	N/A	N/A	N/A	N/A
	12 months	5.03%	0	5.03%	N/A	N/A	0.0014
	24 months	5.03%	0	5.03%	N/A	N/A	0.0014
Abducent nerve palsy	3 months	0	0	N/A	N/A	N/A	N/A
	6 months	0	0	N/A	N/A	N/A	N/A
	12 months	0	0	N/A	N/A	N/A	N/A
	24 months	5.03%	5.03%	0	1	0.426–2.35	0.999
Diplopia	3months	5.03%	5.03%	0	1	0.426–2.35	0.999
	6 months	5.03%	5.03%	0	1	0.426–2.35	0.999
	12 months	5.03%	5.03%	0	1	0.426–2.35	0.999
	24 months	5.03%	5.03%	0	1	0.426–2.35	0.999
Refractory IIH	3 months	30.65%	31.66%	− 1.01%	0.968	0.723–1.297	0.8286
	6 months	40.20%	34.17%	6.03%	1.176	0.91–1.521	0.2133
	12 months	42.71%	40.70%	2.01%	1.049	0.832–1.324	0.6843
	24 months	48.24%	45.23%	3.02%	1.067	0.865–1.316	0.5466
Visual discomfort and visual field defects	3 months	0	5.03%	− 5.03%	N/A	N/A	0.0014
	6 months	5.03%	5.03%	0.00%	1	0.426–2.35	0.999
	12 months	5.03%	5.03%	0.00%	1	0.426–2.35	0.999
	24 months	5.03%	5.03%	0.00%	1	0.426–2.35	0.999
CSF leak	3 months	0	5.03%	− 5.03%	N/A	N/A	0.0014
	6 months	0	5.03%	− 5.03%	N/A	N/A	0.0014
	12 months	0	5.03%	− 5.03%	N/A	N/A	0.0014
	24 months	0	5.03%	− 5.03%	N/A	N/A	0.0014
Therapeutic spinal puncture rate	3 months	0	5.03%	− 5.03%	N/A	N/A	0.0014
	6 months	0	0.00%	−	N/A	N/A	N/A
	12 months	0	5.03%	− 5.03%	N/A	N/A	0.0014
	24 months	5.03%	5.03%	0.00%	1	0.426–2.35	0.999
CSF shunting rate	3 months	0	0	N/A	N/A	N/A	N/A
	6 months	0	0	N/A	N/A	N/A	N/A
	12 months	0	0	N/A	N/A	N/A	N/A
	24 months	0	0	N/A	N/A	N/A	N/A
ONSF rate	3 months	0	0	N/A	N/A	N/A	N/A
	6 months	0	0	N/A	N/A	N/A	N/A
	12 months	0	0	N/A	N/A	N/A	N/A
	24 months	0	0	N/A	N/A	N/A	N/A
Acetazolamide use	3 months	15.08%	12.56%	2.51%	1.2	0.733–1.965	0.4677
	6 months	17.09%	10.05%	7.04%	1.7	1.015–2.849	0.0404
	12 months	20.10%	13.57%	6.53%	1.481	0.948–2.316	0.0816
	24 months	23.12%	14.07%	9.05%	1.643	1.072–2.517	0.0204

#### RYGB versus adjustable gastric banding

The comparison of RYGB and adjustable gastric banding revealed largely comparable outcomes across most parameters (Table 5). Papilledema rates were identical between groups until 24 months, where a non-significant difference emerged (5.10% vs. 6.12%, RR: 0.833, 95% CI: 0.369–1.884, *p* = 0.6607). Headache prevalence showed minimal variation between the two procedures, with the largest non-significant difference observed at six months (41.84% vs. 39.80%, RR: 1.051, 95% CI: 0.828–1.334, *p* = 0.681). Refractory IIH rates were remarkably similar throughout the follow-up period, with risk ratios consistently close to 1.0 (range: 0.99–1.039, all *p* > 0.05). Notably, increased intracranial pressure was observed exclusively in the RYGB group at three and six months (5.10%, *p* = 0.0014 for both), while abducent nerve palsy occurred only in the adjustable gastric banding group from three to 12 months (5.10%, *p* = 0.0014 for each interval). Pulsatile tinnitus manifested solely in the RYGB cohort at 12 and 24 months (5.10%, *p* = 0.0014 for both). Therapeutic spinal puncture was performed only in the adjustable gastric banding group at six and 12 months (5.10%, *p* = 0.0014 for both). Acetazolamide use was consistently higher in the RYGB group across all time points, though the differences did not reach statistical significance (24 months: 22.96% vs. 20.92%, RR: 1.098, 95% CI: 0.755–1.595, *p* = 0.6254). Neither group reported any CSF shunting or ONSF procedures throughout the study duration.

**Table 5:** RYGB versus adjustable gastric banding outcomes comparison.

Outcome	Follow-up duration	RYGB	Adjustable gastric banding	Risk difference	Risk ratio	95% confidence interval	*P*-value
Papilledema	3 months	5.10%	5.10%	0	1	0.426–2.349	0.999
	6 months	5.10%	5.10%	0	1	0.426–2.349	0.999
	12 months	5.10%	5.10%	0	1	0.426–2.349	0.999
	24 months	5.10%	6.12%	− 1.02%	0.833	0.369–1.884	0.6607
Increased ICP	3 months	5.10%	0	5.10%	N/A	N/A	0.0014
	6 months	5.10%	0	5.10%	N/A	N/A	0.0014
	12 months	5.10%	5.10%	0	1	0.426–2.349	0.999
	24 months	5.10%	5.10%	0	1	0.426–2.349	0.999
Headache	3 months	31.12%	30.10%	1.02%	1.034	0.767–1.393	0.8265
	6 months	41.84%	39.80%	2.04%	1.051	0.828–1.334	0.681
	12 months	44.39%	44.39%	0.00%	1	0.801–1.248	0.999
	24 months	50.51%	50.51%	0.00%	1	0.822–1.217	0.999
Optic atrophy	3 months	5.10%	5.10%	0	1	0.426–2.349	0.999
	6 months	5.10%	5.10%	0	1	0.426–2.349	0.999
	12 months	5.10%	5.10%	0	1	0.426–2.349	0.999
	24 months	5.10%	5.10%	0	1	0.426–2.349	0.999
Blindness	3 months	5.10%	5.10%	0	1	0.426–2.349	0.999
	6 months	5.10%	5.10%	0	1	0.426–2.349	0.999
	12 months	5.10%	5.10%	0	1	0.426–2.349	0.999
	24 months	5.10%	5.10%	0	1	0.426–2.349	0.999
Pulsatile tinnitus	3 months	0	0	N/A	N/A	N/A	N/A
	6 months	0	0	N/A	N/A	N/A	N/A
	12 months	5.10%	0	5.10%	N/A	N/A	0.0014
	24 months	5.10%	0	5.10%	N/A	N/A	0.0014
Abducent nerve palsy	3 months	0	5.10%	− 5.10%	N/A	N/A	0.0014
	6 months	0	5.10%	− 5.10%	N/A	N/A	0.0014
	12 months	0	5.10%	− 5.10%	N/A	N/A	0.0014
	24 months	5.10%	5.10%	0	1	0.426–2.349	0.999
Diplopia	3 months	5.10%	5.10%	0	1	0.426–2.349	0.999
	6 months	5.10%	5.10%	0	1	0.426–2.349	0.999
	12 months	5.10%	5.10%	0	1	0.426–2.349	0.999
	24 months	5.10%	5.10%	0	1	0.426–2.349	0.999
Refractory IIH	3 months	30.61%	30.10%	0.51%	1.017	0.753–1.373	0.912
	6 months	40.82%	39.29%	1.53%	1.039	0.815–1.324	0.7571
	12 months	42.86%	42.86%	0	1	0.796–1.257	0.999
	24 months	48.47%	48.98%	− 0.51%	0.99	0.808–1.212	0.9195
Visual discomfort and visual field defects	3 months	0	5.10%	− 5.10%	N/A	N/A	0.0014
	6 months	5.10%	5.10%	0	1	0.426–2.349	0.999
	12 months	5.10%	5.10%	0	1	0.426–2.349	0.999
	24 months	5.10%	5.10%	0	1	0.426–2.349	0.999
CSF leak	3 months	0	0	N/A	N/A	N/A	N/A
	6 months	0	0	N/A	N/A	N/A	N/A
	12 months	0	0	N/A	N/A	N/A	N/A
	24 months	0	0	N/A	N/A	N/A	N/A
Therapeutic spinal puncture rate	3 months	0	0	N/A	N/A	N/A	N/A
	6 months	0	5.10%	− 5.10%	N/A	N/A	0.0014
	12 months	0	5.10%	− 5.10%	N/A	N/A	0.0014
	24 months	5.10%	5.10%	0	1	0.426–2.349	0.999
CSF shunting rate	3 months	0	0	N/A	N/A	N/A	N/A
	6 months	0	0	N/A	N/A	N/A	N/A
	12 months	0	0	N/A	N/A	N/A	N/A
	24 months	0	0	N/A	N/A	N/A	N/A
ONSF rate	3 months	0	0	N/A	N/A	N/A	N/A
	6 months	0	0	N/A	N/A	N/A	N/A
	12 months	0	0	N/A	N/A	N/A	N/A
	24 months	0	0	N/A	N/A	N/A	N/A
Acetazolamide use	3 months	15.31%	9.69%	5.61%	1.579	0.921–2.708	0.093
	6 months	16.84%	13.27%	3.57%	1.269	0.79–2.04	0.3228
	12 months	19.90%	17.35%	2.55%	1.147	0.757–1.737	0.5165
	24 months	22.96%	20.92%	2.04%	1.098	0.755–1.595	0.6254

### BMI longitudinal follow-up analysis

The results of our longitudinal analysis reveal significant differences in BMI outcomes among various weight management strategies over a 24-month follow-up period (Table 6). Bariatric surgery demonstrated superior efficacy compared to community weight management, with a mean BMI difference of -1.3 (95% CI: -2.389, -0.211; *p* = 0.0195) at 3 months, widening to -4.114 (95% CI: -5.060, -3.168; *p* < 0.0001) at 24 months. Among surgical interventions, RYGB initially showed the most substantial BMI reduction, with a mean difference of 3.703 (95% CI: 2.053, 5.353; *p* < 0.0001) compared to sleeve gastrectomy at three months. However, this advantage diminished over time, becoming statistically non-significant by 24 months (mean difference 0.88; 95% CI: -0.870, 2.630; *p* = 0.323). Sleeve Gastrectomy maintained a modest but significant advantage over adjustable gastric banding at six months (mean difference − 1.704; 95% CI: -3.046, -0.362; *p* = 0.013), though this difference also became non-significant by the 24-month follow-up. Notably, while all surgical interventions resulted in sustained BMI reductions over the study period, the rate of BMI decline slowed considerably after the first year post-surgery.

**Table 6:** Body mass index comparison over different follow-up time points.

Month	Bariatric surgery		Community weight management		Mean difference	95% confidence interval	*P*-value
	Mean BMI	SD	Mean BMI	SD			
3 months	37.43	9.293	38.73	9.6	− 1.3	(− 2.389, − 0.211)	0.0195
6 months	35.95	8.8	38.136	9.549	− 2.186	(− 3.189, − 1.183)	< 0.0001
12 months	34.627	8.64	38.314	9.516	− 3.687	(− 4.647, − 2.727)	< 0.0001
24 months	34.087	8.627	38.201	9.636	− 4.114	(− 5.060, − 3.168)	< 0.0001

Month	Sleeve gastrectomy		Adjustable gastric banding		Mean difference	95% confidence interval	*P*− value
3 months	37.678	7.999	39.077	7.952	− 1.399	(− 2.828, 0.030)	0.055
6 months	35.58	7.81	37.284	7.765	− 1.704	(− 3.046, − 0.362)	0.013
12 months	34.515	8.045	35.631	8.279	− 1.116	(− 2.472, 0.240)	0.106
24 months	34.013	8.062	35.025	8.756	− 1.012	(− 2.385, 0.361)	0.148

Month	RYGB		Sleeve gastrectomy		Mean difference	95% confidence interval	*P*− value
3 months	41.749	7.007	38.046	7.693	3.703	(2.053, 5.353)	< 0.0001
6 months	38.718	6.855	35.738	8.043	2.98	(1.310, 4.650)	0.0005
12 months	35.945	7.179	34.209	8.053	1.736	(0.045, 3.427)	0.044
24 months	34.667	7.351	33.787	8.488	0.88	(− 0.870, 2.630)	0.323

Month	RYGB		Adjustable gastric banding		Mean difference	95% confidence interval	*P*− value
3 months	41.908	7.046	39.799	8.005	2.109	(0.355, 3.863)	0.018
6 months	38.825	6.885	37.665	7.982	1.16	(− 0.508, 2.828)	0.172
12 months	36.03	7.203	35.841	7.99	0.189	(− 1.500, 1.878)	0.826
24 months	34.731	7.413	34.74	7.804	− 0.009	(− 1.681, 1.663)	0.992

### Safety analysis

Post-operative complications within 30 days of bariatric surgery included wound infections (3.2%), anastomotic leak (1.1%), and pulmonary complications (2.4%). No mortality was reported in the immediate post-operative period. Long-term complications included nutritional deficiencies requiring supplementation (15.7% at 12 months) and need for revisional surgery (2.8% over 24 months).

## Discussion

Our study provides compelling evidence for the efficacy of bariatric surgery in managing IIH, demonstrating superior outcomes compared to conventional community weight management approaches. These findings contribute to the growing body of literature supporting surgical interventions for IIH, particularly in patients with concurrent obesity. The significantly lower incidence of papilledema in the bariatric surgery group across all follow-up periods is a key finding of our study^16^. The sustained reduction in papilledema rates over 24 months in our cohort suggests that bariatric surgery may offer a more durable solution for managing this critical manifestation of IIH. This is particularly noteworthy given the potential for permanent vision loss associated with chronic papilledema^17^.

Our results also demonstrated a consistent reduction in headache prevalence among bariatric surgery patients. This finding corroborates the Yri et al.^17^ evidence who reported improvements in headache severity and frequency following weight loss in IIH patients^17,18^. The mechanism underlying this improvement likely involves a combination of reduced intracranial pressure and alleviation of comorbid conditions associated with obesity, such as obstructive sleep apnea, which can exacerbate IIH symptoms^18^. Interestingly, our results demonstrated a higher incidence of blindness in the bariatric surgery group at 12 months post-intervention. This unexpected finding warrants careful interpretation. It may reflect a subset of patients with more advanced disease at baseline, who were more likely to be referred for bariatric surgery. Alternatively, it could be related to rapid weight loss following surgery, which has been associated with transient worsening of visual symptoms in some IIH patients^12^. This underscores the importance of close ophthalmological monitoring in the post-operative period and highlights an area for future research to elucidate the relationship between rapid weight loss and visual outcomes in IIH.

The reduced prevalence of visual discomfort and visual field defects in the bariatric surgery group at 12 and 24 months is encouraging. These findings suggest that surgical weight loss may offer more comprehensive protection against the visual sequelae of IIH compared to conventional weight management. This aligns with the work of Fridley et al.^12^, who reported improvements in visual acuity and visual field parameters following bariatric surgery in IIH patients^12^. The lower rates of acetazolamide use in the bariatric surgery group, particularly at 12 and 24 months, suggest a reduced need for pharmacological intervention post-surgery. This finding has important implications for long-term management and quality of life, given the significant side effect profile associated with acetazolamide^10^.

Moreover, the absence of ONSF procedures in the bariatric surgery group is noteworthy, potentially indicating a reduced need for invasive interventions to manage refractory cases. Our subgroup analysis comparing different bariatric procedures yielded several intriguing findings. The lack of significant differences in most outcomes between sleeve gastrectomy and adjustable gastric banding suggests that both procedures may be viable options for IIH management.

However, the trend towards lower acetazolamide use in the sleeve gastrectomy group at 24 months, although not statistically significant, warrants further investigation. The comparison between RYGB and sleeve gastrectomy revealed largely comparable outcomes, with some notable exceptions. The exclusive observation of increased intracranial pressure in the RYGB group at three months is intriguing and may reflect differences in the early post-operative course between these procedures. The significantly higher acetazolamide use in the RYGB group at six and 24 months is also noteworthy and merits further exploration to understand the underlying mechanisms.

Our longitudinal BMI analysis demonstrated the superior efficacy of bariatric surgery in achieving sustained weight loss compared to community weight management. This finding is consistent with large-scale studies on bariatric surgery outcomes in the general obese population^19^. The initial advantage of RYGB over sleeve gastrectomy in BMI reduction, which diminished over time, aligns with recent meta-analyses comparing these procedures^20^. The slowing of BMI decline after the first year post-surgery underscores the importance of long-term follow-up and support for bariatric patients to maintain weight loss.

It is important to note that our study did not include biliopancreatic diversion with duodenal switch (BPD/DS) or single-anastomosis duodeno-ileal bypass with sleeve gastrectomy (SADI-S) procedures, as no IIH patients in the TriNetX database underwent these surgeries during our study period. This limitation reflects the relative rarity of these procedures compared to RYGB, sleeve gastrectomy, and adjustable gastric banding^21^. While surgical intervention carries inherent risks, the complication rates observed were acceptable and should be weighed against the potential benefits of sustained weight loss and IIH symptom improvement. A significant limitation of our study is the inability to conduct cost-effectiveness analysis, as the TriNetX platform does not provide healthcare cost data. Future studies incorporating detailed economic analysis would be valuable in comparing the long-term financial implications of bariatric surgery versus conventional management for IIH patients. This analysis should include not only direct surgical costs but also long-term expenses related to medication use, follow-up care, and management of complications.

Also, the emergence of GLP-1 receptor agonists represents a significant development in obesity management that may impact future treatment algorithms for IIH. While our study period preceded the widespread adoption of these agents, recent evidence suggests their potential role in IIH management^22–26^. These medications achieve significant weight loss through appetite suppression and delayed gastric emptying, potentially offering an alternative or bridge to bariatric surgery in selected patients. Future studies comparing bariatric surgery to GLP-1 agonist therapy in IIH patients will be crucial in establishing optimal treatment pathways.

Future research incorporating these more complex malabsorptive procedures could provide valuable insights into their potential role in IIH management. Our study has several strengths, including its large sample size, propensity score matching to minimize confounding, and comprehensive outcome assessment. The use of real-world data from the TriNetX network enhances the generalizability of our findings.

However, we acknowledge several limitations. First, as an observational study, we cannot establish causality. Second, the retrospective nature of our analysis may introduce selection bias, despite our efforts to mitigate this through propensity score matching. Third, the TriNetX database, while extensive, may not capture all relevant clinical details, potentially leading to underreporting of some outcomes. Moreover, our study did not account for potential differences in surgical technique or post-operative care protocols across institutions, which could influence outcomes. Additionally, we were unable to assess the impact of bariatric surgery on cerebrospinal fluid dynamics or venous sinus stenosis, both of which are implicated in IIH pathophysiology^3^. Future prospective studies incorporating neuroimaging and intracranial pressure monitoring could provide valuable insights into the mechanisms underlying the observed improvements. In addition to that, the reliance on ICD coding for diagnosis and outcome assessment may have led to underreporting of certain symptoms, particularly subtle changes in papilledema or visual fields. The database structure did not allow for detailed assessment of disease severity or duration at baseline, which could have influenced treatment outcomes through floor or ceiling effects. Additionally, the retrospective nature of our study meant that visual field testing and papilledema assessment were not standardized across centers, potentially affecting the reliability of these outcome measures.

## Conclusions

Our findings provide new evidence supporting the efficacy of bariatric surgery in managing IIH, demonstrating superior outcomes compared to conventional weight management approaches across multiple parameters. The sustained improvements in papilledema, headache, and visual symptoms, coupled with reduced need for pharmacological and surgical interventions, suggest that bariatric surgery may offer a more definitive solution for carefully selected IIH patients with concurrent obesity. However, the unexpected finding of increased blindness rates at 12 months post-surgery underscores the need for vigilant post-operative monitoring and highlights an important area for future research. The comparable efficacy of different bariatric procedures in our study suggests that the choice of surgical technique may be tailored to individual patient factors and surgeon expertise. As the prevalence of obesity continues to rise globally, the incidence of IIH is likely to increase correspondingly. Our results contribute to the evolving paradigm of IIH management, suggesting that early consideration of bariatric surgery in appropriate candidates may improve long-term outcomes and reduce the burden of chronic medical therapy. Future studies should focus on developing evidence-based guidelines for patient selection, optimizing post-operative care protocols, and elucidating the mechanisms underlying the observed improvements in IIH symptoms following bariatric surgery.

## Data Availability

The utilized data was obtained from TriNetX platform without direct access to individual data level, the accessibility to data analysis shall be obtained from the platform directly.

## Declarations

### LLM statement

We have employed an advanced Large Language Model (LLM) to enhance and refine the English-language writing. This process focused solely on improving the text’s clarity and style, without generating or adding any new information to the content.

### Statement of methods compliance

All the used methods in this retrospective cohort study were carried out in accordance with relevant guidelines and regulations, including the Declaration of Helsinki and Good Clinical Practice guidelines for retrospective database research.

### Institutional Review Statement

This retrospective study utilizing de-identified data from the TriNetX Research Network was determined to be exempt from review by the Albert Einstein College of Medicine Institutional Review Board as it does not constitute human subjects research according to 45 CFR 46.104(d)(4).
